# Audiological findings in patients treated with radio- and concomitant chemotherapy for head and neck tumors

**DOI:** 10.1186/1748-717X-4-53

**Published:** 2009-11-15

**Authors:** Ana Helena B Dell'Aringa, Myrian L Isaac, Gustavo V Arruda, Maria Carolina BN Esteves, Alfredo Rafael Dell'Aringa, José Luis S Júnior, Alexandre F Rodrigues

**Affiliations:** 1 Departament of Otorhinolaryngology of FAMEMA's General Hospital, Marília, Sao Paulo, Brazil; 2 Departament of Otorhinolaryngology of FMUSP- RP, Ribeirao Preto, Sao Paulo, Brazil; 3 Departament of Oncology, Radiotherapy of FAMEMA's General Hospital, Marília, Sao Paulo, Brazil; 4 FAMEMA, Marília, Sao Paulo, Brazil; 5 Marília, Sao Paulo, Brazil

## Abstract

**Objective:**

To evaluate the functionality of the auditory system in patients who underwent radiotherapy and chemotherapy treatment with cisplatin to treat head and neck tumors.

**Study Design:**

Case series with planned data collection.

**Setting:**

From May 2007 to May 2008 by the Department of Otorhinolaryngology and the Department of Oncology/Radiotherapy at *Faculdade de Medicina de Marília*.

**Subjects and Methods:**

Audiological evaluation (Pure Tone Audiometry (air and bone conduction), Speech Audiometry, Tympanometry, Acoustic Reflex testing and Distortion Product Otoacoustic Emissions) was performed in 17 patients diagnosed with head and neck neoplasia and treated with chemotherapy, using cisplatin, and radiotherapy.

**Results:**

12 left ears (70.5%) and 11 right ears (64.7%) presented bilateral decreased hearing soon after the treatment for the frequency 1 kHz (mild auditory damage) and for the frequency 8 kHz (more significant auditory damage).

**Conclusion:**

Patients with head and neck cancer submitted to the conventional radiotherapy treatment, combined with the chemotherapy with cisplatin, presented a high incidence of decreased hearing by the end of treatment. Strong evidence was observed linking auditory alteration to the amount of radiotherapy treatment.

## Introduction

Auditory damage is one of the main complications of oncological therapy in patients with head and neck tumors [1]. Recently, the addition of chemotherapy (CT) with cisplatin to radiotherapy (RT) has been improving the survival rate of patients with these neoplasias, becoming a standard treatment for tumors locally advanced. However, both treatments, chemotherapy with cisplatin and radiotherapy, are known for their ototoxic effects.

As the combined treatment became the standard procedure in many cases of head and neck tumors, the objective of this study is to evaluate the changes in auditory function in patients submitted to these procedures regarding their ototoxic effects; to describe the incidence of the precocious auditory damages after the combined treatment; to establish a link between the findings obtained and the factors related to the treatment, including the cumulative dose of chemotherapy, the total dose of radiotherapy, and the volume irradiated by the radiotherapy (VTRT).

## Methods and materials

Case series with planned data collection performed by the Department of Otorhinolaryngology and the Department of Oncology/Radiotherapy at *Faculdade de Medicina de Marília *(FAMEMA), approved by the Research Ethics Committee under protocol # 165/06.

The study involved 17 patients (15 males and 2 females, in a total of 34 ears) that were scheduled for chemoradiation treatment for extracranial head and neck tumors, from May 2007 to May 2008. All patients agreed to undergo hearing tests and all provided written informed consent for participation in this study.

After having the diagnosis of neoplasia confirmed by the anatomopathological examination, and being indicated this combined treatment, the patients were directed to the Otorhinolaryngology clinic at FAMEMA's General Hospital (*Hospital das Clínicas*) for otorhinolaryngological and audiological evaluation, which consisted of two stages: pre- and post-treatment (within 2 weeks after treatment). In these stages, the following procedures were used: Anamnesis and Clínical Otorhinolaryngological Evaluation, Audiological Anamnesis, Pure Tone Audiometry by air and bone conduction, Speech Audiometry, Tympanometry, Acoustic Reflex testing and Distortion Product Otoacoustic Emissions (DPOAE).

Patients with otitis media with effusion, hearing loss due to a significant noise exposure, trauma, ototoxic medication, neoplasm, and congenital infection or syndrome, congenital or pediatric onset, with nasopharyngeal tumors and those who previously received chemo and/or radiotherapy were excluded from the study.

In the Department of Oncology/Radiotherapy, all patients underwent a complete physical examination, direct or indirect laryngoscopy, head and neck computerized tomography and thorax X-ray before the treatment.

Weekly doses of intravenous cisplatin (30 mg/m^2^) as first infusion of the drug in the first day of the radiotherapy was prescribed to the patients.

This prescribed radiotherapy dose varied according to the primary site of the disease and the primary tumor staging. Before beginning the radiotherapy sessions, the patients were submitted to the conventional simulation (X-ray) for delimitation of the radiotherapy site and to make molded thermoplastic masks, used to immobilize the patient in supine position and to mark the area to be treated.

The treatment limits (radiation area) varied according to the primary site of the pathology. However, due to the advanced nature of the cases, all the patients had the superior border of the radiotherapy site in the base of the cranium, thus including the cochlea in the radiotherapy area.

To calculate the volume of radiotherapy treatment (VRT) we used the patient's latero-lateral distance, measured during the simulation process, multiplied by the resulting area of the simulation area for each patient.

### Statistical analysis

To analyze the differences in the tonal threshold averages for each frequency, pre and post treatment, we performed the T - Student Test.

To determine which values (variables: age, volume and dose of RT and total dose of CT) would have some relation to the reduction of the tonal thresholds in the post-treatment, the Fisher Exact Test was applied. According to the Contingency table, the Odds Ratio was calculated to determine the relation between the probability of occurrence to the probability of non-occurrence of an auditory alteration after chemo- and concomitant radiotherapy treatment, taking into consideration the variables: age, volume and dose of RT, and total dose of CT.

We considered as reduction of the auditory acuity, the decrease of 20 dB in an isolated frequency or of 10 dB in two or more successive frequencies, according to the ASHA criteria [2].

For all the statistical tests, a value up to 5% for the significance level (value of p < 0.05) was considered.

## Results

The patient's characteristics including age, gender, tumor histology, distribution by clinical staging, number of chemotherapy cycles, cumulative dose of cisplatin, fractioned and total dose of radiotherapy, and the volume of radiotherapy treatment are described on Table 1.

**Table 1:** Demonstrates data referring to gender, age, tumor localization, distribution by clinical staging, number of chemotherapy cycles, cumulative dose of cisplatin, dose by fraction and total dose of radiotherapy and the volume of radiotherapy treatment (n = 17).

Gender	Age	Localization	Staging Clínico	Number of cycles QT	Total Dose QT (mg)	Fraction Dose RT (Gy)	Total Dose RT (Gy)	VRTT (cm^ 3^)
M	54	CEC Larynx	T4 N2 M0	7	336	1.8	70.2	1,390
M	49	CEC Larynx	T3 N3 M0	5	300	1.8	70.2	2,028
M	71	CEC Hypopharynx	T4 N2 M0	5	270	1.8	70.2	2,364
M	51	CEC Hypopharynx	T2 N2 M0	7	315	1.8	70.2	1,664
M	61	CEC Oropharynx	T4 N2 M0	8	400	1.8	70.2	1,480
M	55	CEC Oral Cavity	T2 N2 M0	8	336	1.8	72.2	1,550
M	69	CEC Hard Palate	T3 N1 M0	6	270	1.8	70.2	1,306
M	51	CEC Unseen	Tx N2 M0	8	220	1.8	70.2	739.64
M	75	CEC Base Tongue	T3 N3 M0	7	350	1.8	70.2	2,460
M	69	CEC Oropharynx	T4 N0 M0	2	96	2	68	1,392
F	40	CEC Larynx	T4 N0 M0	7	455	2	72	1,614
M	57	CEC Larynx	T4 N0 M0	6	258	1.8	70.2	1,488
M	64	CEC Larynx	T3 N3 M0	7	364	2	64	2,258
M	58	CEC Hypopharynx	T4 N3 M0	3	144	1.8	70.2	1,754
F	68	CEC Unseen	Tx N2 M0	7	378	2	60	3,364
M	70	CEC Oral Cavity	T2 N1 M0	7	399	2	64	1,728
M	54	CEC Oropharynx	T3 N0 M0	4	192	1.8	70.2	2,925

All the patients received at least 2 chemotherapy cycles, and three patients received 8 cycles resulting in an average dose of 299 mg/m^2 ^and median of 315 mg/m^2 ^with variation from 96 to 455 mg/m^2^.

The total dose of the radiotherapy varied from 60.0 Gy to 72.0 Gy, being the average dose of 68.9 Gy and median of the dose of 72.2 Gy, with a variation of the daily dose from 1.8 Gy to 2.0 Gy.

Regarding the VRT, we have found an average volume of 1,853 cm^3 ^and median of 1,500 cm^3 ^with variation of 739 cm^3 ^- 3,364 cm^3^.

Out of 17 patients and 34 ears analyzed, we observed during the otorhinolaryngological evaluation that 2 ears presented perforated tympanic membrane and 32 normal membranes. Alterations during the chemo and radiotherapy treatment were not observed in the otoscopy.

Regarding the audiological evaluations performed before the radio and chemotherapy treatments, it was observed in our sample that only 2 ears presented thresholds within the normal patterns (lower than 20 dB) and most of the patients analyzed had already presented some type of auditory alteration: 3 ears with mixed hearing loss (one with a mild hearing loss and 2 with a moderate hearing loss).

The other 29 ears presented sensorineural hearing loss of unknown etiology: 4 ears showed mild sensorineural hearing loss, 10 ears exhibited sensorial hearing loss at frequencies above 1 KHz, 10 ears had hearing loss above 2 KHz and 5 ears showed hearing loss at frequencies above 3 KHz.

The pre treatment hearing loss etiology may have had a clinical diagnosis of presbycusis because of the age of the patients. Patients that had known causes of hearing loss were excluded as described in Methods.

**Figure 1:**
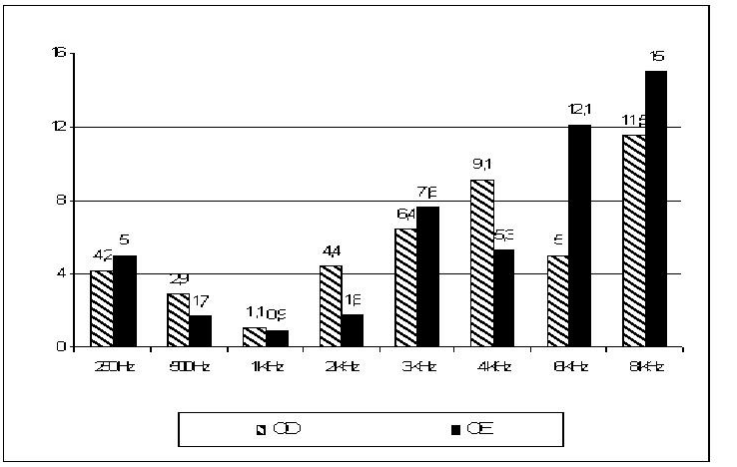
**Demonstrates the average differences between pre and pos-treatment air conduction hearing thresholds in decibel, for the frequencies ranging from 250 HZ to 8 KHz, in left and right ears, for the 17 patients analyzed**.

In the analysis of the data obtained, it was possible to verify that the average of the frequencies analyzed from 0.25 kHz to 8 kHz were shown to be significantly overset when comparing pre- and post-treatment, as shown on figure 1 and Table 2, which also shows data referring to the standard deviation, confidence interval and p-value for each frequency bilaterally.

**Table 2:** Demonstrates the difference between the averages of air conduction tonal thresholds pre and post-treatment by frequency by ear, standard deviation, confidence interval and p-value.

	Frequence	Diference between the pré- and post-treatment average	Standard Deviation	95% Diference Confidence Interval		p-Valor
				Inferior Limit	Superior Limit	
LEFT EAR	250	-2.94	4.69	-5.35	-0.52	0.02
	500	-2.94	3.97	-4.98	-0.89	0.008
	1 KHz	-1.17	3.32	-2.88	0.53	0.163
	2 KHZ	-4.41	7.68	-8.36	-0.46	0.031
	3 KHz	-6.47	11.42	-12.34	-0.59	0.033
	4 KHz	-9.11	9.22	-13.86	-4.37	0.001
	6 KHZ	-5.00	8.47	-9.35	-0.64	0.027
	8 KHz	-11.47	9.31	-16.25	-6.68	0.00
						

						
RIGHT EAR	250	-5.00	5.30	-7.72	-2.27	0.001
	500	-1.76	3.03	-3.32	-0.20	0.029
	1 KHz	-0.88	2.64	-2.24	-0.47	0.188
	2 KHZ	-1.76	4.30	-3.98	-0.45	0.111
	3 KHz	-7.64	8.12	-11.82	-3.47	0.001
	4 KHz	-5.29	4.83	-7.77	-2.8	0.00
	6 KHZ	-12.05	11.59	-18.02	-6.09	0.001
	8 KHz	-15.00	14.03	-22.21	-7.78	0.00

According to the ASHA criteria, described above, 70.5% (12 left ears) and 64.7% (11 right ears) presented decreased hearing soon after the treatment.

The frequencies of 1 kHz and 8 kHz presented smaller and larger auditory alteration (respectively), bilaterally.

The cumulative dose of chemotherapy > 300 mg/m^2^, the dose by cycle > 50 mg/m^2 ^and the total dose of radiotherapy > 70.2 Gy did not show association with decreased hearing, according to Fisher exact test statistical analysis. The only factors associated to this decreased hearing, according to the ASHA criteria were the age ≤ 60 years (p = 0.046) and VTRT > 1500 cm^3 ^(p = 0.016) (Table 3).

**Table 3:** Demonstrates the relation between the probability of occurrence to the probability of non-occurrence of an auditory damage after chemoradiotherapy treatment, being taken into consideration the variables age, volume and dose of RT and total and cycle dose of CT.

		Age		Total	p-Valor
		≤ 60 years	> 60 years		
Auditory damage	Yes	9	4	13	0.046
ASHA	No	7	14	21	
**Total**		**16**	**18**	**34**	

		**RT Volume**		**Total**	**p-Value**
		**≤ 1500 cm**	**> 1500 cm**		
Auditory alteration	Yes	8	5	13	0.016
ASHA	No	4	17	21	

**Total**		**12**	**22**	**34**	

		**RT Total Dose**		**Total**	**p-Value**
		**≤ 70.2 Gy**	**> 70.2 Gy**		
Auditory damage	Yes	3	10	13	0.182
ASHA	No	5	16	21	

**Total**		**8**	**26**	**34**	

		**CT Dose Total**		**Total**	**p-Value**
		**≤ 300 mg**	**> 300 mg**		
Auditory damage	Yes	7	6	13	0.393
ASHA	No	9	12	21	

**Total**		**16**	**18**	**34**	

		**CT Dose per Cycle**		**Total**	**p-Value**
		**≤ 50 mg**	**> 50 mg**		
Auditory damage	Yes	9	4	13	0.272
ASHA	No	11	10	21	

**Total**		**20**	**14**	**34**	

Results about EOAE-DP and acoustic reflexes were analyzed but they did not show statistical significance. EOAE-DP were absent in most patients before treatment. In others, was not possible to realize this exam due to large noisy respiration.

## Discussion

The Radiotherapy is one of the most effective treatments for head and neck tumors.

For initial lesions T1 and T2, the results of exclusive use of RT are comparable to the ones obtained using surgical procedures. For lesions in a more advanced degree, as some head and neck anatomical sites, RT associated to CT has been preferred, due to the possibility of preserving the organ. The complications of the radiotherapy are specific sites, in other words, they depend on the area (radiotherapy field) in which the radiation is being applied.

Specifically for the advanced disease, due to the presence of voluminous tumors, there is a need to irradiate the primary site and suspicious areas of microscopic disease. A potential gamma of complications mainly to healthy tissues close to the tumoral layer may appear as a consequence. As an example, we can mention ototoxicity in cases of RT on the head and neck area.

Our study observed an important correlation between VRT and auditory acuity. (Table 3)

In spite of not having performed RT by 3-D image, we have estimated the VRT for each patient using the technical parameters of simulation and treatment. It was not possible to estimate the exact amount of radiation dose received by the cochlea. However it was observed that the patients that received VRT >1,500 cm^3 ^were the ones that presented more auditory damage.

The probable explanation for this discovery is that the patients that received VRT > 1,500 had the cochlea irradiated with doses above 45 Gy. This fact, possibly, has led these patients to receive radiation doses in the cochlea between 50 and 60 Gy, while patients that received VRT < 1,500, after this dose (45 Gy), had the cochlea or part of it out of the RT field.

Few studies have evaluated the effect of the volume of irradiated treatment and the relationship of the total dose of RT received by the cochlea with its irradiated volume, as a prognostic factor for the reduction of auditory acuity.

What is known is the relationship between the punctual dose of radiation received by the cochlea when the three-dimensional conformed equipment of RT is used, as shown by Chen et al., who evaluated 22 patients submitted to chemo-radiation [3]. They observed that in the frequencies of 3,000 and 2,000 Hz, the mean dose of radiotherapy > 48 Gy and time (12 months) were significant in multivariate and univariate analyses, whereas the total dose of cisplatin was not shown to have a statistically significant contribution to the change in hearing thresholds.^3 ^In that study, a statistically significant contribution from cochlear mean dose (Dmean) was seen, but not cisplatin, suggesting that after more than12 months post-treatment the radiation dose may supersede cisplatin in affecting long-term sensorineural sequelae. The ototoxic effect of radiotherapy found in our study, shows the relationship between the radiotherapy treatment volume and Dmean in cochlea. Patients irradiated with VRT > 1,500 cm^3 ^probably received total doses of radiotherapy higher than 48 Gy in cochlea. Data suggest that the total dose of radiation may supersede cisplatin in a short follow-up.

Regarding the treatment dose of radiotherapy, results of our statistical analysis do not show significance for the decreased hearing post-treatment in patients that received doses higher than 70.2 Gy of radiation. It is important to highlight that this radiation dose is the total area dose of the performed treatment and not the dose received by the cochlea.

A recent study reported that sensorineural hearing loss is a potential complication after RT due to cochlear damage. Herrman et al observed significant reduced hearing ability, starting with high frequencies at 40 Gy, subsequently progressing to deeper frequencies at 60 Gy and post-RT [4].

Other studies that compared patients treated only with radiotherapy and patients treated with chemo and concomitant radiotherapy describe higher risk of hearing loss in the second group [5,6]. Therefore, we cannot rule out the possibility of a cumulative effect and potentiation of the combined treatment.

The ototoxicity caused by cisplatin happens acutely, being its collateral effects seen in the first days after its use. The audiological changes are typically bilateral, irreversible and progressive; they begin in high frequencies with subsequent extension for medium and low frequencies as the number of cycles increases [6].

Some reports show that when the cumulative dose of cisplatin reaches 240 mg/m^2^, a significant loss of the high frequencies starts to happen, and the ototoxity increases as the accumulated dose increases. Others show 400 mg/m^2 ^as a damaging dose [7,8].

Ho et al. reported that the mean dose of 275 mg/m^2 ^of cisplatin was not associated to the increase of hearing threshold [9]. That result is in accordance with our data. The dose of cisplatin used in our group of patients was relatively low (96-455 mg/m2, mean dosage 299 mg). Our statistical analysis, did not show significant correlations between the total CT dose and the increase of the hearing thresholds, being the average and the median dose 300 mg/m^2 ^(p = 0.393). (Tables 3)

The age of patients submitted to the treatment has also been evidenced as a factor in the increase of the auditory acuity reduction risk [10,11].

In our study, all the patients analyzed presented age ≥ 40 years old, (average age of 60 years old). In the statistical analysis, it was verified that the patients < 60 years old presented 4.54 times higher chance to increase hearing ability after the treatment.

Regarding the alteration in the hearing thresholds soon after the end of the chemo- and radiotherapy treatment, we have found in our sample a larger increase of the hearing thresholds for frequencies from 3 KHz to 8 KHz, for both left and right ears.

All patients included presented auditory damage: 70.5% of them with significant auditory alteration; 55.8% with change equal or higher than 20 dB in a unique frequency (first ASHA criterion for ototoxity); and 14.7% with reduction of 10 dB in at least two consecutive frequencies (second ASHA criterion for ototoxity).

These data are in accordance with the results of Pearson et al., who retrospectively evaluated audiological findings in 15 patients with head and neck tumors, treated with radio- and concomitant chemotherapy, using the same criteria to define ototoxicity. Their results showed that 85% of patients presented changes in frequencies from 4 to 8 kHz and more than 50% of them presented a change equal to or higher than 10 dB [1].

Other studies illustrate that auditory deficiency happens in 9% to 91% of the patients, usually bilateral and initially in the high frequencies (4.000-8.000 Hz), also affecting the medium and low frequencies with extended treatment. The auditory deficiency may present a certain degree of reversibility, when the auditory deficiency is not deep [12,13].

In a prospective study, Ho et al. evaluated 526 ears of patients with nasopharyngeal cancer treated exclusively with radiotherapy [9]. Within a 4.5-year period of follow up, they observed that the auditory alterations began soon after the end of the radiotherapy. After two years, 40% of the patients partially recovered the auditory alteration, while the other 60% presented worsening auditory deficiency year after year.

Regarding the auditory complaints presented by the patients, many studies have reported appearance of tinnitus. Some of them report that the tinnitus is usually transitory, disappearing some hours or weeks after the end of the treatment in 2% to 36% of the patients [12,13]. In a recent study performed by Zocoli et al., 46% of the patients presented tinnitus after the second cisplatin application, which remained until the end of the treatment [14].

In our study, two patients complained about tinnitus during the treatment but it disappeared after the end of it. Two complained about otalgia throughout treatment, and one presented descamative otitis externa, also throughout treatment.

## Conclusion

Patients with head and neck cancer submitted to conventional radiotherapy treatment combined with the chemotherapy, presented a high incidence of decreased hearing by the end of the treatment.

The main factor associated with this auditory damage was the volume of radiotherapy treatment.

Our data highlighted the importance of making the auditory evaluation, pre and post-treatment, in all patients submitted to conventional radiotherapy treatment combined with chemotherapy with cisplatin for head and neck tumors.

Thus, we stress the importance of the side effects consequences of the treatment, so that the patients can be early inserted in programs of auditory rehabilitation.

## Final considerations

All the patients that presented auditory degradation in frequencies responsible for speech audibility were led to the use of hearing aids. The three patients who reported daily auditory difficulties and interest in using hearing aids were directed and enrolled in the Hearing Aid Program of this Institution.

## Competing interests

The authors declare that they have no competing interests.

## Authors' contributions

AHBD carried out the data collection and design of the study, MLI carried out of the design and coordination of the study, GVA carried out of the data collection, design and statistical analysis, MCBNE carried out of the data collection, ARD carried out of the design and coordination of the study, JLSJ carried out of the data collection ad English review, AFR carried out of the data collection ad English review. All authors read and approved the final manuscript.
